# To be only human: Humanizing effect of lowering humanity

**DOI:** 10.1192/j.eurpsy.2021.847

**Published:** 2021-08-13

**Authors:** S.L. Poe

**Affiliations:** Psychology, The New School, New York, United States of America

**Keywords:** Social Psychology, Dehumanization, Social Comparison, Moral Psychology

## Abstract

**Introduction:**

The derogation and violence associated with describing others as less than human is documented in a wide range of research (e.g., Bandura, 1992; Optow, 1990). However, this research has only explored one side of the social comparisons that humanity can evoke. Integrating dehumanization research and that of social comparison, which suggests the different effects of upward and downward targets, we explore social comparison which lowers human nature and therefore raises the target (Suls et al., 2002; Suls et al., 2018).

**Objectives:**

While dehumanization places others below humanity, we explore hyper-humanization which places humanity below other comparison classes. When humanity is characterized as a low social comparison class, this should lead people to reintegrate transgressors, evoke forgiveness and reduce revenge motives.

**Methods:**

To test this hypothesis 577 participants viewed a vignette about a social transgression and completed the benevolence and avoidance subscales of the TRIM (McCullough et al., 2006). We manipulated dehumanization and hyper-humanization using a conversational prompt which asked participants to elaborate on descriptions of the social transgressor. In the dehumanization condition the target was described as “barely human”, and in the hyper-humanization condition the target was described as “only human”.

**Results:**

Using a mixed-model ANOVA, results indicate that in the rehumanization condition benevolence increased (F=5.30, p<.01) and avoidance decreased (F=4.75, p<.01) relative to dehumanization and controls.

**Conclusions:**

While lower groups below humanity may facilitate genocide and other social ills, other forms of social comparison with humanity may act to restore relationships and facilitate forgiveness.

